# Costs and outcomes of colorectal cancer screening program in Isfahan, Iran

**DOI:** 10.1186/s12913-022-09010-1

**Published:** 2023-01-05

**Authors:** Farimah Rahimi, Reza Rezayatmand, Javad Shojaeenejad, Elham Tabesh, Zahra Ravankhah, Peyman Adibi

**Affiliations:** 1 Health Management and Economics Research Center, Isfahan University of Medical Sciences, Isfahan, Iran; 2 Department of Health Economics, School of Management and Medical Information Sciences Isfahan University of Medical Science, Isfahan, Iran; 3 Isfahan Gastroenterology and Hepatology Research Center (IGHRC), Isfahan University of Medical Sciences, Isfahan, Iran; 4 Cancer Registry of Health Deputy, Isfahan University of Medical Sciences, Isfahan, Iran

**Keywords:** Colorectal cancer, Screening, Direct costs, Outcomes

## Abstract

**Background:**

Colorectal cancer is one of the most prevalent gastrointestinal cancers in Iran i.e., the fourth and the second prevalent cancer among Iranian males and females, respectively. A routine screening program is effective in the early detection of disease which can reduce the cancer burden both for individuals and society. In 2015, Iran’s Package of Essential Non- communicable Diseases program had been piloted in Shahreza city in Isfahan province. Colorectal cancer screening for the population aged 50–70 was a part of this program. So far, there was no study about the cost and outcomes of that program. Thus, this study aimed to analyze the costs and outcomes of colorectal cancer screening done from 2016 to 2019 in Shahreza.

**Methods:**

This cost-outcome description study used the data of 19,392 individuals who were 50–70 years old experienced a fecal immunochemical test (FIT) and had an electronic health record. All direct costs including personnel, building space, equipment, training, etc. were extracted from the financial documents existing in the Isfahan province Health Center. The outcome was defined as positive FIT, detection of adenoma or malignancy as recorded in the E-integrated health system.

**Results:**

The results of this study indicated that the direct costs of the colorectal cancer screening program during the years 2016–2019 were 7,368,707,574 Rials (321,029 PPP$) in Shahreza, Isfahan province. These costs resulted in identifying 821 people with a positive FIT test, of those 367 individuals were undergone colonoscopy. Of whom 8 cases of colorectal cancer, and 151 cases with polyps were diagnosed.

**Conclusion:**

This study showed that by paying a small amount of 320 thousand international dollars we could prevent 151 cases of polyps to be progressed to colorectal cancer,resulting in a significant reduction in colorectal cancer incidence.

## Background

Cancer has caused 9.9 million deaths in the world in 2020 and it could be considered a significant public health problem [1]. Coming after lung cancer, colorectal cancer (CRC) is reported as the second most common cause of cancer-related death in 2020 in the world [1]. CRC has been ranked as fourth cancer among Iranian males after gastric, prostate, and lung cancers and the second one among Iranian females, after breast cancer, [1]. Studies have indicated sufficient evidence about the effectiveness of colorectal cancer screening as one of the best and most worthwhile methods for early diagnosis of disease [2]. A common screening strategy in many countries is doing the fecal immunochemical test (FIT) and then following positive results with a colonoscopy. One study by Arrospide et al., screened people at age 50 to 69 in Spain by FIT. They estimated the total cost of screening, diagnostic follow-up, surveillance, and treatment at €2057 million for 3 years (2005–2008). However, as the consequence, it was shown that the screening program reduced the incidence of colorectal cancer as well as the costs of treatment and increased the life expectancy of those who participated in the screening program compared to those who did not participate in the screening program [3]. In another study, the total direct cost of a 3-years comprehensive colorectal cancer prevention program was estimated at $261 per person [4].

Accordingly, the prevention and control of non-communicable diseases can reduce premature mortality and disability significantly. In order to realize this objective, the National Document on Prevention and Control of Non-Communicable Diseases and Related Risk Factors in the Islamic Republic of Iran was developed. The first phase of Iran’s Package of Essential Non- communicable Diseases (IraPEN) program that evaluated the risk of non-communicable diseases began in 2015 in the primary health center of four cities (Baft, Naghadeh, Maragheh, and Shahreza). In Shahreza, located in Isfahan province, IraPEN started in February 2015 [5]. CRC screening was an important component of IraPEN.

All people who are 50 to 70 years old were called to the primary health center where they were registered in the E-integrated health system (SIB (in Persian)) in terms of any cancer-related symptoms or familial history. Then, they were trained and asked to do a FIT test at home. Based on the FIT result, people were being followed. Those with negative FIT were asked to do a test every 2 years, and those with positive FIT were referred for colonoscopy [6].

Since the implementation of the CRC screening program, as the first program in Iran, the cost and consequence of the program had not been studied yet. Thus, this study aimed to report and analyze the cost and consequence of the program since started. So, this study had been conducted to examine the costs and outcomes of the colorectal cancer screening program during 2016–2019 in Shahreza, Isfahan province.

## Methods

This was a Cost-consequence analysis (CCA) that described the costs and outcomes of the CRC screening program in Shahreza in the period of 2016–2019. CCA is one type of economic evaluation study that compares the costs and outcomes of a program. Unlike other methods of economic evaluation, this method does not try to compare the cost and outcomes of a program with other alternatives. This method analyzes the costs and outcomes of a program simply by using descriptive tables to help the experts and policymakers to make a better decision regarding the question if the intervention or program is worthy or not [7].

### Screening program

In the pilot CRC screening program run in Shahreza, Isfahan, a mix of active/ non-active inviting strategies was used. To invite eligible people, a public call was made through city-wide notification by using media, local radio, and television and distributing banners. In addition, people who had gone to the health center to receive other services were asked to participate in the program. In general, those eligible people who did not participate had been invited to be screened by a maximum of three phone calls.

### Outcomes

All registered people in the E-integrated health system (SIB (in Persian)) in Shahreza city who were 50–70 years old and have taken the FIT test entered into this study. The outcomes were identified as adherence rate (the number of participants/ all 50–70 years people), positive rate (the number of positive tests/ the number of FITs), colonoscopy adherence rate (the number of positive FIT tests followed by colonoscopy/ all positive tests), polyp detection rate (the number of patients with polyp(s)/ all colonoscopy cases), and CRC detection rate (the number of patients with cancer/ all colonoscopy cases).

The required data such as the number of positive FITs, the number of colonoscopies in positive FITs, the number of detected people with polyps, and the number of detected cancer cases were collected from Isfahan province Health Center and Shahreza Health Center.

Descriptive analysis such as frequency and mean was used to describe outcome data. Screening outcomes were described via frequency and rates, and data analysis was done in Excel for Windows.

### Costs

Direct costs of the CRC screening program included the costs of inviting the target group and the cost of implementing the program that was gathered based on the health care perspective. So, we categorized the costs of the colorectal cancer screening program into seven categories i.e., personnel; buildings and space; equipment; supplies, and pharmaceuticals; transportation; training; and social mobilization and publicity [8]. Table 1 shows more details of this categorization.

**Table 1:** Costs categories, practical definitions, and their sources

Cost groups:	Including	Source
personnel	Including salaries, fees paid to the gastroenterologist, general practitioners, health care professionals, nurses, and other personnels for FIT testing, patient referrals, and colonoscopy.	Financial documents relating to the IraPEN program.
buildings and space	Price of the colonoscopy clinic building. Estimating Building maintenance and depreciation costs and utility costs (water, electricity, gas, telephone) for 2016–2019	Documents related to land purchase and review of bills of energy carriers and depreciation and maintenance of colonoscopy clinic equipment.
equipment	Cost of buying a colonoscopy set, catheter, suction, pulse oximeter, resuscitation trolley, medical bed, patient bed, monitoring device, DC shock device.	Invoice for purchasing equipment of medical and hospital equipment unit.
Supplies and pharmaceuticals	Cost of KIT, syringe, distilled water, angiocath, disposable clothing and sheets, Droshite, latex gloves, surgical gloves, lidocaine gel, forceps, snare, plates, clips, and injection needles. Senagraf syrup, Pidrolax powder, Bisacodyl tablets, Dimethicone tablets, Midazolam ampoule.	Purchase invoice for consumable equipment performing FIT test and colonoscopy available in hospital pharmacy.
training	Cost of personnel involved in training all caregivers in health centers for colorectal cancer screening.	financial documents related to the training fee of teaching staff.
transportation	Cost of transporting supplies, pharmaceuticals and personnel.	Financial documents relating to the IraPEN program.
social mobilization and publicity	The information and advertising costs of the program in the form of banners, pamphlets, tracts, and placards.	

The cost data were collected from the accounting department of the Isfahan province Health Center and Shahreza Health Center as well as the accounting department of Amir Al-Momenin Hospital. Staff involved in the program management were also interviewed when necessary. All calculated costs are reported as present value (2019) in Rials and also Dollars that are adjusted by the year-specific Purchasing Power Parity (PPP) conversion factor reported by the World Bank’s International Comparison Program indicators [9]. The program costs were calculated in Excel software.

## Results

About 19,335 individuals were eligible for the program and about 28% of the target population were invited to the screening program annually. Finally, all the target population was invited for 4 years. The mean participation rate was 26% annually which ranged between 16 to 44%. This study included the result of the first screening round only. During the study period, a total of 19,392 people participated in the CRC screening program in Shahreza, of which about 53% were female. As depicted in Table 2, the mean age of participants was 56.6 ± 6.1and the level of education of about 72% was elementary school or less. 99.5% of the participants had Iranian nationality and only 0.5% had non-Iranian nationality. Also, 0.05% of screened people (9 persons) had reported individual adenoma history in themselves and about 1% (196) had reported colorectal cancer history in first or second-degree relatives.

**Table 2:** Characteristics of The CRC screening participants

Variable		Number	Percentage
Target population	2016	17,500	
	2017	18,285	
	2018	19,351	
	2019	22,204	
	Total/Average	19,335	
Invited population	2016	9079	52%
	2017	3978	22%
	2018	3206	17%
	2019	4466	20%
	Total/Average	20729	
Number of participants	2016	7764	44%
	2017	3975	22%
	2018	3187	16%
	2019	4466	20%
	Total/Average	19,392	100%
Gender	Female	10,256	52.9%
	Male	9136	47.1%
Nationality	Iranian	19,286	99.5%
	Non-Iranian	106	0.5%
Education	illiterate	5075	26.2%
	Elementary	8864	45.7%
	High School	4362	22.5%
	University	1091	5.6%
History of CRC or adenoma	No history	19,187	98.98%
	The individual history of adenoma	9	0.05%
	History of CRC in 1st-degree relatives	163	0.84%
	History of CRC in 2nd-degree relatives	33	0.17%

### Outcomes

From 2016 to 2019, overall, 19,392 FIT tests were performed. Of those 821 (4.2%) were positive. 367 (45%) individuals with the positive FIT test had been followed by colonoscopy, resulting in finding 8 cases of CRC as well as 151 cases of polyps. So, the average detection rates were 40 and 2% for polyp and CRC cases, respectively. As depicted in Table 3, the number of reported polyp cases increased during these 4 years while the highest number of undertaken colonoscopies and detected cancer was seen in 2018.

**Table 3:** Outcomes of the colorectal cancer screening program

Consequences:		2016	2017	2018	2019	Total/Average
Positive FIT test	Number	282	108	146	285	821
	Positive rate	3.63%	2.72%	4.58%	6.38%	4.23%
colonoscopy	Number	67	89	129	82	367
	Colonoscopy adherence rate	23.76%	82.41%	88.36%	28.77%	44.70%
Polyp cases	Number	14	26	53	58	151
	Polyp detection rate	20.90%	29.21%	41.09%	70.73%	41.14%
Cancer cases	Number	4	1	2	1	8
	CRC detection rate	5.97%	1.12%	1.55%	1.22%	2.18%

### Costs

The total cost of the CRC screening program in Shahreza from 2016 to 2019 was about 7,368,707,574 Rials based on the Consumer price index (CPI) indicator in 2019 (321,029 PPP$). More than half of all costs happened in the first year of the program by 4,342,202,632 Rials (186,073 PPP$). As shown in Table 4, about 38% of all costs were personnel costs by 2,788,392,708 Rials (123,053 PPP $). If the cost of staff training were also added, the personnel cost would consist of about 64% of all costs. The cost of equipment, pharmaceutical, and building consisted of 20, 8, and 6% of all costs, respectively.

**Table 4:** Direct costs of the colorectal cancer screening program

Cost of	values	2016	2017	2018	2019	Total
**Personnel**	Present value (Rials)	719,456,999	641,003,413	512,428,234	915,504,062	2,788,392,708
	Present value (PPP$)	30,830	27,013	23,738	41,472	123,053
	%					38%
**Buildings and space**	Present value (Rials)	391,984,954	5,538,789	5,317,277	4,569,200	407,410,220
	Present value (PPP$)	16,797	233	246	207	17,484
	%					6%
**Equipment**	Present value (Rials)	1,051,507,910	175,277,804	171,272,349	72,832,620	1,470,890,683
	Present value (PPP$)	45,059	7387	7934	3299	63,679
	%					20%
**Supplies and pharmaceuticals**	Present value (Rials)	164,389,762	104,090,999	144,984,768	175,742,900	589,208,429
	Present value (PPP$)	7044	4387	6716	7961	26,108
	%					8%
**Training**	Present value (Rials)	1,918,041,158				1,918,041,158
	Present value (PPP$)	82,192				82,192
	%					26%
**Transportation**	Present value (Rials)	84,232,911	34,433,086	34,472,943	29,036,500	182,175,440
	Present value (PPP$)	3610	1451	1597	1315	7973
	%					2%
**Social mobilization**	Present value (Rials)	12,588,938				12,588,938
	Present value (PPP$)	539				539
	%					0%
**Total**	Present value (Rials)	4,342,202,632	960,344,091	868,475,571	1,197,685,282	7,368,707,576
	Present value (PPP$)	186,073	40,471	40,231	54,254	321,029
	%	59%	13%	12%	16%	100%

**Fig. 1:**
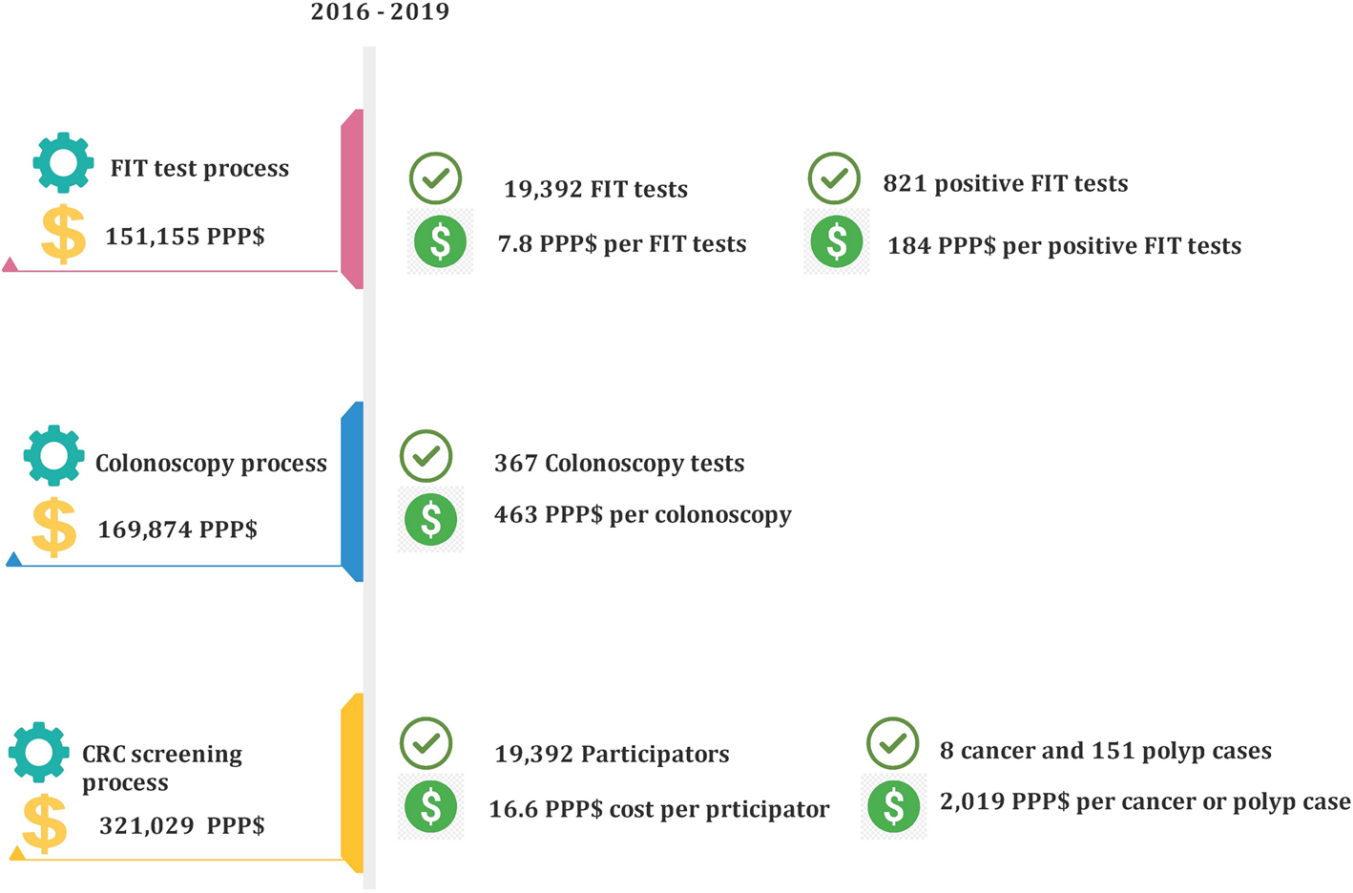
Cost and Outcomes of CRC screening program in Shahreza, Iran, 2016–2019

The results showed that the total cost for each screened person in the CRC screening program in Shahreza was 379,987 IRR (16.6 PPP$). If the ultimate goal of the CRC screening program was to find any case of polyp or cancer, a cost of 46,344,073 IRR (2019 PPP$) had been paid for each detection of polyp or cancer case. Excluding the cost of colonoscopy and the cost of capital investment in the first year of the program, the FIT test process had 151,155 PPP$ costs that it means the health system faced 7.8 $ for every FIT test. Also, the health system costs an average of 462.9 $ for each colonoscopy performed. Figure 1 is a representation of this information:

## Discussion

This was a cost-outcome description study that aimed to describe and analyze the cost and outcomes of the CRC screening program which had been piloted in Shahreza city in Isfahan province from 2016 to 2019 as an integrated part of the IraPEN program. In this study, we looked at what was done in the CRC screening program, not what should have been done. The study found that of 19,392 individuals who had undertaken FIT, 821 individuals had a positive result. Of those, 367 were followed by colonoscopy, resulting in finding 151 cases of polyp(s) and 8 cases of cancer. For every FIT test, an amount of 180,087 Rials (7.8 PPP$) had been paid by the health system. For every detected polyp or cancer case, the system incurred an amount of 46,344,073 Rials (2019 PPP$).

To compare the results of this study with the previous studies, it should be noted that few studies have reported the direct costs of CRC screening programs in such detail as our study. Most related studies calculated the screening cost based on a model such as Csanádi, et al. (2020) [10], Lew. Et al. (2017) [11] and Tangka et al. (2017) [12]. But implementation of the Mailed FIT program in Washington State showed that the total cost per FIT kit returned was about $40 (including planning costs), and $19 (only implementation costs) [13]. In one study by Lansdorp-Vogelaar et al. (2017) a unit cost of €7 (8$) for FIT (including kit and analysis only, excluding organizational costs) was reported [14]. However, for any comparison, different costing methods should be taken into account. The Lansdorp-Vogelaar et.al calculation for the cost of the FIT kit was based on the Medicare reimbursement rate while in our study the price was taken from the market. It should be noted that the price of consumables and the FIT kit was increasing during the study period. In another model-based study, Allameh et al. (2009), which aimed to assess the cost-effectiveness of selected colorectal cancer screening methods in Iran on 100,000 people between 45 and 65 years old, the cost of each Fecal Occult Blood Test (FOBT), sigmoidoscopy, and CT colonography for diagnosis of a patient; The cost of different screening strategies and treatment of the patient was 0.22, 0.28 and 0.42 billion Rials in the public sector and 1.68, 1.54 and 60.1 billion Rials in the private sector, respectively in twenty years [15]. However, as this study only included the costs of treatment, and valued the costs based on the tariff and not the real cost, it seemed not to be comparable to our study calculating the actual costs of the implementation of the CRC screening program.

Concerning reported outcomes, we found that on average in the 4 years about 4.2% of screened individuals, in a range of 2.7 to 6.4% in different years, has positive FIT results. That it is lower than the reported positive in Tehran, Iran (9.2%) [6], Nigeria (20.5%) [16], Uruguay (11.1%) [17], Brazil (9.7%) [18], and Thailand (8.7%) [19] in the average-risk population and near to Mexico (5.9%) [20]. The mean proportion of FIT-positive results ranged from 8% by using the OC-Micro test to 21% for Hemosure [21]. But the desired rate of positive FIT is determined as 5% by the Iranian Ministry of Health based on the announced instructions. The difference between this rate and others can be caused by the type of used Kit in Shahreza, Iran, which needs to be studied in another research.

Regarding the percentage of those followed by colonoscopy, on average, about 45% of those with positive FIT were undergone colonoscopy. However, it ranged from 24 to 88% in different years. The lowest rates were related to the first and last years of the program which can be explained by the lack of access to colonoscopy and not preparing the system in the first year and also decreasing the staff sensitization and complete follow-up in the last year. Studies reported lack of knowledge, fear of the result, procedure and pain, lack of awareness, high cost, and lack of gastrointestinal symptoms as common barriers [22].

The polyp detection rate and CRC detection rate in the study among Iranian patients were 23.5 and 1.5%, respectively [23]. When comparing our results, we found mean cancer and polyp detection rates about 2 and 41% respectively, which is higher than the results of the Asadzadeh Aghdaei et al. study. It might be because that study performed a cross-sectional retrospective study, including individuals aged 15 to 85 years, who underwent their first colonoscopy during 2014–2015.

To generalize the results to the province and the country, it should be considered that we do not have any evidence showing that the population of Shareza city is more willing to participate in the screening program. Thus, we can assume that this population is representative of the whole country. Even if it’s not the case, we do not have evidence that the detection rate is correlated with the participation rate, meaning that those who are more willing to participate are more susceptible to having polyp or cancer. Regarding the generalization of costs, because the personnel costs, which contain the largest share of whole costs, are valued almost the same throughout the country, there is less concern about the generalizability of costs. However, for those areas with lesser participation, it would be expected that the cost per person diagnosed with cancer or polyps to be a bit higher than those areas with more participation due to the effect of fixed costs.

The reason that we could not conduct a full economic evaluation was the fact that we did not have any information on the effectiveness of the CRC program in Shahreza. Thus, this study was designed as a cost-consequence analysis. By using our findings in addition to effectiveness data, further research can be conducted to analyze the cost-effectiveness of the program.

### Limitation

Due to data limitations, we faced some limitations. The way people had been invited was not recorded so the participation rate cannot be examined in different inviting strategies. Also, the data was recorded for only one round so it was not possible to compare the participation rate in different rounds. Another limitation was that we could not differentiate adenoma polyps from other polyps. Also, since this screening was done in the public sector, only public sector costs were included in the study.

## Conclusion

In this study, we use real financial data to provide a more precise estimation of the cost of the screening program which can be used in future model-based studies. This study showed that by paying a little amount of 321,029 $ we can prevent 151 cases of polyps to be progressed to CRC.

## Data Availability

All data generated and analyzed during this study are included in this published article.

## Abbreviations

CRC: Colorectal cancer
IraPEN: Iran’s Package of Essential Non- communicable Diseases
FIT: Fecal immunochemical test
CCA: Cost-consequence analysis
PPP: Purchasing Power Parity
CPI: Consumer price index
